# An Open SCADA-Based Supervisory Architecture for an Experimental Reverse Osmosis System: Development and Functional Validation

**DOI:** 10.3390/s26196058

**Published:** 2026-09-24

**Authors:** Daniel Antonio Oliveira Santana, Ruben Barros Godoy, Tiago Henrique de Abreu Mateus, Keila Roberta Ferreira de Oliveira

**Affiliations:** 1 Graduate Program in Electrical Engineering, Faculty of Engineering, Architecture and Urbanism and Geography, Federal University of Mato Grosso do Sul, Campo Grande 79070-900, MS, Brazil; ruben.godoy@ufms.br (R.B.G.); tiago.mateus@ufms.br (T.H.d.A.M.); 2 Faculty of Engineering, Architecture and Urbanism and Geography, Federal University of Mato Grosso do Sul, Campo Grande 79070-900, MS, Brazil; keila.ferreira@ufms.br

**Keywords:** Industrial Internet of Things (IIoT), edge computing, supervisory control and data acquisition (SCADA), MQTT, sensor-state validation, reverse osmosis, alarm management, functional validation, remote configuration

## Abstract

Recent studies demonstrate growing interest in supervisory systems based on open architectures, but existing implementations report fragmented combinations of functions and validation evidence. This study developed and functionally validated an open four-layer SCADA-based supervisory architecture for an experimental reverse osmosis (RO) system. Eleven commercial off-the-shelf sensors were integrated through ESP32-based acquisition, MQTT communication, a Raspberry Pi/Flask edge server, SQLite persistence, and a browser-based human–machine interface (HMI). Validation combined historian analysis, controlled functional scenarios, bidirectional configuration tests, service-recovery trials, and a 600 s edge-resource assessment. The historian contained 190,840 records covering all eleven tags, with values, states, units, and timestamps. Controlled tests verified coherent measurement-state processing, alarm persistence and acknowledgment, thermal-dependency handling, stopped-state logic, diagnosis, and HMI presentation. Valid configuration changes were confirmed by the ESP32, whereas an out-of-range coefficient was rejected without changing the active value. All backend, broker, communication-path, and edge-server recovery trials met predefined criteria, with median recovery times of 6.384, 1.312, 1.517, and 90.604 s, respectively. During the controlled workload, 6600 MQTT publications and 480 HTTP requests were processed without errors, and SQLite integrity was preserved. The results demonstrate integrated cross-layer operation under the tested conditions. The architecture did not implement automatic closed-loop control of pumps or valves. Metrological performance, cybersecurity, usability, and scalability were outside the validation scope.

## 1. Introduction

The digital transformation associated with Industry 4.0 has deeply changed the organization of industrial automation systems. Cyber–physical systems (CPSs), the Industrial Internet of Things (IIoT), data analytics, and distributed computing have increased the need for interoperability between field devices, control systems, supervisory platforms, and management applications. In this context, supervisory control and data acquisition (SCADA) systems have evolved from predominantly centralized and monolithic solutions toward architectures that support distributed components, web technologies, and standardized communication interfaces [1,2].

This revolution has also affected water-treatment systems, which require continuous and simultaneous monitoring of hydraulic, physicochemical, and operational variables [3,4]. In reverse osmosis (RO), variables such as pressure, flow rate, temperature, pH, and electrical conductivity (EC) are used to follow process operation and the condition of the produced water [4,5]. Although periodic laboratory analyses and local measurements remain relevant, their discontinuous nature limits the immediate identification of operational changes. Continuous supervision allows process conditions and operational events to be recorded, supporting historical analysis, operation, and decision making [6].

Previous studies have demonstrated the feasibility of open and distributed technologies for the supervision of reverse-osmosis and desalination systems [5,7,8,9,10,11]. However, the examined implementations report different combinations of acquisition, communication, historical storage, alarm management, human–machine interaction, operational diagnosis, and remote configuration. The research gap addressed in this work is the absence, among the reviewed publications, of an open SCADA-based supervisory architecture that reports the integration and functional validation of this complete set of services within a single platform. This observation concerns the functions and validation evidence documented in the reviewed publications and does not imply that the corresponding architectures are technically incapable of incorporating additional services. Therefore, the aim of this study is to develop and validate an open SCADA-based supervisory architecture for an experimental RO system that integrates the services required for process monitoring, supervision, and operational management. To achieve this goal, the functional and architectural requirements of the platform were defined, the proposed architecture was designed, and mechanisms for distributed data acquisition and communication between the field devices and the supervisory platform were developed. The monitoring, historical data persistence, alarm management, human–machine interface, operational diagnosis, and remote-configuration modules were then implemented and integrated into a single platform.

The specific contribution of this study is the integrated implementation and functional validation, under defined test conditions, of this set of supervisory services within a single open four-layer architecture. The contribution concerns the reported combination of functions and validation evidence rather than the isolated use of the selected hardware, protocols, or software components. The scope is supervisory: the architecture acquires, processes, stores, diagnoses, and presents process information and supports remote configuration of measurement parameters, but it does not execute automatic closed-loop control of pumps, valves, pressure, or flow rate.

The resulting architecture was evaluated through complementary evidence obtained from the instrumented experimental RO system and through a controlled set of software tests of the implemented supervisory functions. The validation verified compliance with the established functional requirements and whether the implemented modules operated coherently as parts of the proposed architecture.

## 2. Theoretical Background and Related Work

### 2.1. Domain-Specific Characteristics of Reverse-Osmosis Systems

Osmosis is a natural phenomenon characterized by the spontaneous transport of a solvent through a semipermeable membrane from a solution with a lower solute concentration to one with a higher solute concentration. The osmotic pressure is the minimum pressure required on the more concentrated solution to prevent this spontaneous solvent flow. Its magnitude depends on the concentration and nature of the dissolved species and on the solution temperature, rather than being an intrinsic property of the membrane [12].

Reverse osmosis (RO) is a pressure-driven membrane-separation process in which an external pressure greater than the osmotic pressure forces water through a semipermeable membrane while retaining a substantial fraction of the dissolved solutes. The process produces two principal streams: permeate, with a lower concentration of dissolved salts, and concentrate, also referred to as retentate or brine, in which the retained species become more concentrated [13]. RO desalination can be applied to feedwater from aquifers, rivers, seas, and industrial processes. Data reported for 2020 indicate that approximately 85% of the active desalination plants worldwide employed RO technology and that approximately 90% of the units under construction were designed to use it [14].

The monitoring of pressure, flow rate, temperature, pH, and electrical conductivity (EC) is critical in RO desalination because these variables are directly related to process performance, membrane condition, and the quality of the produced water [4]. Their interpretation requires considering both the hydraulic behavior of the system and the physicochemical conditions of the feed, membrane-feed, permeate, and concentrate streams [15,16].

Operating pressure affects both solute rejection and energy consumption. Under otherwise constant feedwater conditions, increasing the applied pressure generally increases the apparent solute rejection and permeate production. However, higher pressures also increase energy demand and may contribute to membrane compaction, thereby reducing membrane service life [15,16]. In addition to absolute pressure, the pressure difference between process regions provides information about hydraulic resistance. A progressive increase in differential pressure can be associated with material deposition and deterioration of membrane performance [17].

Temperature influences solute solubility, diffusion coefficients, permeate flux, and salt passage through the membrane. Consequently, variations in feed temperature can modify the measured conductivity of the streams and the apparent separation performance of the system. Temperature measurements are therefore required not only as direct indicators of operating conditions but also to contextualize the interpretation of the other process variables [15,16].

The pH of the process streams is relevant to membrane performance and to the formation of inorganic deposits. Depending on the feedwater composition, pH may intensify scaling through the precipitation and deposition of sparingly soluble salts on the membrane surface. The rejection performance of polyamide membranes also varies with pH, with maximum solute rejection generally occurring within a limited operating range [15,16]. Accordingly, pH measurements contribute to the characterization of the acid–base condition of the feed and permeate streams and to the identification of operating conditions that may affect membrane integrity.

Electrical conductivity and total dissolved solids are indicators of the ionic content of water and consequently of the separation performance of an RO system. Comparing feedwater and permeate conductivity enables the assessment of ionic rejection, whereas an increase in permeate conductivity may indicate deterioration of the produced-water quality or changes in membrane performance [16]. Conductivity measurements should therefore be interpreted jointly with temperature, pressure, and flow-rate measurements rather than as isolated indicators.

Flow-rate measurements characterize the hydraulic distribution of the process. The feed, permeate, and concentrate flow rates support the determination of the hydraulic balance and performance indicators such as system recovery. An increased feed flow rate may also reduce concentration polarization at the membrane surface by limiting the local accumulation of solutes [15,16]. The simultaneous observation of pressure and flow rate is consequently necessary to distinguish changes in hydraulic operating conditions from changes in separation performance.

Most RO plants therefore employ instrumentation to monitor these variables, which provide the basis for historical analysis and operational decision-making [16]. Pressure, differential pressure, and flow rates characterize the hydraulic regime and membrane behavior; EC and ionic rejection characterize separation performance and permeate quality; pH indicates the acid–base condition of the streams; and temperature contextualizes the behavior of the other measurements. Supervising an RO system consequently requires continuous data acquisition, contextual interpretation of measurements, identification of abnormal conditions, and temporal persistence of the acquired information. Together, these functions allow process measurements to be converted into information about system performance and operating condition.

### 2.2. Open Supervisory Architectures and Functional Requirements

Open supervisory architectures seek to reduce the technological dependence, limited interoperability, and restricted adaptability associated with proprietary systems. Architectural openness is not defined exclusively by the use of open-source software or low-cost hardware, but by modularity, interoperability, scalability, portability, and reduced vendor dependence. These properties are particularly relevant to experimental applications, in which instrumentation and operating conditions may change throughout the system life cycle [3,11,18,19].

Modularity separates data acquisition, communication, processing, persistence, and visualization into relatively independent functional units. Interoperability allows heterogeneous sensors, embedded devices, servers, and software applications to exchange information through documented protocols, interfaces, and data formats. Scalability and portability, in turn, support the incorporation or replacement of measurement and computational components without requiring the complete reconstruction of the supervisory system [3,11,18,19].

The human–machine interface (HMI) is defined as the set of hardware and software through which an operator interacts with the controller of a machine or process. ANSI/ISA-101.01-2015 addresses the development, implementation, operation, and maintenance of HMIs, including visual standardization, hierarchical display organization, consistent navigation, human factors, and ergonomics. The standard organizes process displays into four levels: Level 1 provides an overview of the main process parameters, alarms, and conditions; Level 2 comprises the primary displays used for routine operation and normal monitoring; Level 3 provides detailed system information for non-routine operations and diagnosis; and Level 4 provides detailed information about individual points, support procedures, and safety interlocks. This hierarchy allows the operator to progress from an overall representation of the process to progressively more detailed information [20].

Alarm management provides operational meaning to abnormal process conditions. IEC 62682:2022 defines an alarm as an indication of an abnormal condition requiring a specific operator response, distinguishing it from events and informational messages. Alarm interpretation must consider the operating state because a condition requiring intervention during normal operation may be expected during startup, shutdown, or maintenance. Acknowledgment (ACK) indicates that the operator has recognized the occurrence but does not necessarily eliminate the originating condition. Process condition, alarm state, and operator acknowledgment must therefore remain distinguishable throughout the alarm life cycle [21].

The reliability of supervisory functions depends on whether the acquired data adequately represent the physical process. Instrumentation failures, communication losses, missing records, and invalid measurements may produce numerical values that are unsuitable for operational interpretation. Relevant data-quality properties include accuracy, completeness, consistency, timeliness, and validity, while corresponding mechanisms include range validation, identification of missing or invalid values, detection of acquisition failures, and association of quality states with measurements [22]. Each value must also retain a timestamp representing its temporal reference because communication and processing delays may cause records to arrive late or out of order [23]. Data provenance complements temporal consistency by relating a stored record to its source and to the transformations performed during acquisition, transmission, processing, and storage [24].

Historical persistence is traditionally assigned to the industrial historian, which stores information acquired from the physical process and supports trend visualization, reconstruction of operational occurrences, and performance analysis [25]. Persistence alone, however, does not ensure reliable historical information because invalid values, inconsistent timestamps, or records without identifiable origin may remain unsuitable for interpretation. Data quality, temporal consistency, traceability, and persistence are therefore complementary properties of the supervisory-data life cycle [3,22,24].

### 2.3. Related Work

The supervisory architectures applied to desalination processes differ in the distribution of data acquisition, communication, processing, visualization, persistence, and control functions. Alshehri et al. [8] proposed a distributed IoT architecture in which sensors and embedded controllers communicate through Zigbee, while Wi-Fi connects the system to a private-cloud infrastructure. The cloud portal provides remote visualization, analysis, alerts, and data storage. Uddin et al. [5], in contrast, implemented an open SCADA architecture for a community solar-powered RO system using field devices, an RTU, and an MTU connected through serial communication. Node-RED, Grafana, and InfluxDB were hosted locally in the MTU, concentrating supervision, analysis, and historical persistence in a local computational unit.

Kafrashi et al. [10] addressed operation without Internet or mobile-network connectivity by separating the architecture into field and operation units connected through LoRa. ESP32 devices performed field acquisition and exchanged data and commands with a local Mosquitto MQTT broker and a FUXA interface. The architecture supported bidirectional control and was evaluated through seven laboratory scenarios. Seoudy et al. [26] examined centralized and decentralized architectures for the Nuweiba SWRO plant, with a capacity of 15,000 m^3^/day. Both configurations retained an industrial hierarchy comprising field devices, PLCs, SCADA servers, operator stations, and an industrial communication network. Their comparison showed that decentralization affected not only the system topology but also scalability, availability, maintenance, cabling, and infrastructure costs.

Poyatos-Bakker et al. [11] developed an open-software SCADA system for a demonstrative membrane-distillation plant. FUXA provided the web supervisory interface, control applications operated in Docker containers, MQTT and OPC UA supported communication, and SQLite provided local persistence. This arrangement demonstrated functional separation at the software level. Laredj et al. [27] proposed the DigiRO framework, comprising process, network, control, and supervision layers. A Python 3.11.2 physical model represented the RO process, virtual sensors exposed its variables through Modbus registers, a PLC implemented the control layer, and a SCADA interface provided supervision. The model was validated using more than 1000 h of industrial data and presented a maximum relative error of 6%.

No predominant supervisory architecture can be identified among these studies. Some proposals concentrate processing, storage, and visualization in a local master unit, whereas others distribute these functions among field devices, operation stations, cloud services, containers, or industrial control layers. Despite the diversity of technologies, the studies show a tendency toward functional separation and the use of open or standardized communication interfaces [5,8,10,11,26,27].

The implemented functions and validation strategies also differ. Some platforms emphasize acquisition and visualization, whereas others incorporate remote control, alarms, analytical services, or digital process models. Measurement validation, communication-failure detection, retained-value identification, and treatment of inconsistent data receive limited attention, while historical-persistence mechanisms are explicitly documented in only part of the reviewed architectures. Validation was performed using experimental prototypes, laboratory benches, demonstrative plants, industrial installations, or digital models; consequently, differences in objectives, environments, and evaluation criteria prevent direct comparison of the reported results [5,8,10,11,26,27].

The literature therefore demonstrates progress toward open, modular, and distributed supervision of desalination processes. Nevertheless, the integration of equipment-state visualization, historical storage, event recording, and alarm management within a single open supervisory platform remains limited. This limitation defines the gap addressed by the architecture developed and functionally validated in this study.

## 3. Materials and Methods

### 3.1. Experimental Reverse Osmosis System

The proposed supervisory architecture was implemented and validated in an experimental reverse osmosis (RO) system developed within the Água Dura Project at the Federal University of Mato Grosso do Sul (UFMS) [28]. The system was originally designed to treat groundwater with elevated hardness and salinity toward potable-water production, particularly for small communities affected by limited access to suitable drinking water. In this study, the experimental unit constituted the physical process connected to the supervisory architecture, providing the process measurements and operating states used in its functional validation.

The hydraulic system comprised a pretreatment train, a feedwater tank, a multistage centrifugal pump, an RO separation unit and interconnecting piping. Raw water passed through four filters connected in series: a 5 μm polypropylene cartridge, a zeolite filter, a 50 μm pleated polypropylene cartridge, and an activated-carbon block cartridge. After pretreatment, the water was stored in the feedwater tank and subsequently supplied to the RO separation unit by the centrifugal pump. The low-pressure sections were constructed using 3/4 in PVC piping, whereas the pressurized line connecting the pump to the RO unit used 1/2 in CPVC piping. The pump had a nominal power rating of 3 kW and a maximum flow rate of approximately 1546 L h^−1^. The RO separation unit contained a 4 in × 40 in polyamide/polysulfone membrane element with a maximum rated operating pressure of 42 bar and a nominal permeate flow rate of 441 L h^−1^. The pressurized feedwater was separated by the membrane into permeate and concentrate streams. The hydraulic arrangement of the experimental system and the locations of the measurement channels are shown in Figure 1.

Eleven measurement channels, covering five measured quantities, were distributed along three monitored process sections. The upstream process section, identified by the 1xx numerical series, comprised pressure, flow rate, temperature, pH, and EC measurements. The membrane-feed line, represented by the 2xx series, comprised pressure and flow-rate measurements. The permeate line, represented by the 4xx series, included flow rate, temperature, pH, and EC measurements. The concentrate line was not directly instrumented.

The instrument letter codes followed ANSI/ISA-5.1-2024 guidelines [29]. The first letters P, F, T, and A denoted pressure, flow, temperature, and analysis as the measured variables, respectively. The succeeding letter T denoted the transmitter function. Table 1 summarizes the measurement channels and their roles in process supervision.

The measurement channels were implemented using commercial off-the-shelf (COTS) devices: Water Pressure Sensor G1/4 transducers, YF-S201 flow sensors, DS18B20 temperature sensors, PH-4502C pH modules, and Keystudio TDS Meter V1.0 modules, which were employed for EC measurement. These devices, which were experimentally calibrated before the supervisory validation, were selected according to their measurement ranges, commercial availability and electrical and signal-interface compatibility with the ESP32. Low-cost sensors are recurrent in IoT-based water-quality monitoring literature. However, their accuracy and reliability remain application dependent [30]. For this reason, the devices were used as process-data sources for the functional validation of the supervisory architecture rather than as reference-grade instruments. The temperature channels were used both for direct process monitoring and for temperature compensation of the pH and electrical-conductivity measurements.

The physical implementation of the measurement channels is shown in Figure 2. The photograph identifies the instrument groups, signal-conditioning interfaces, and ESP32-based acquisition node installed in the experimental system.

The experimental installation provided the physical process and measurement channels used for data acquisition and historical-data assessment. The scope of the implemented architecture was supervisory: it comprised measurement acquisition, data communication, visualization, alarm management, historical persistence, diagnostic presentation, and remote configuration of sensor parameters. The system did not implement automatic closed-loop control of the pump, valves, pressure, or flow rate. Accordingly, the validation addressed the integration and functional behavior of the supervisory services rather than the regulatory-control performance of the reverse-osmosis process.

### 3.2. Functional and Architectural Requirements

The supervisory platform specification considered the research objective, the operational characteristics of the experimental RO system, and principles identified in the literature on distributed and open SCADA architectures [1,2,5,10,11]. The requirements were established before implementation. To distinguish architectural verification from functional validation, the seven architectural requirements were assigned the identifiers AR1–AR7, whereas the three functional requirements were assigned FR1–FR3. The ARs constrained the allocation of responsibilities, component organization, and interfaces. They were assessed primarily by inspection of the implemented architecture. The FRs defined services whose operation could be evaluated experimentally. Table 2 lists the ten system-level requirements and their main design implications.

AR1–AR3 and AR5–AR7 were verified by inspecting the implemented allocation of responsibilities, module interfaces, communication mechanisms, technology stack, and four-layer organization. AR4 expressed the intended extensibility of the architecture but was not experimentally evaluated by adding new tags, field devices, or supervisory modules; consequently, no quantitative scalability claim is made. FR1, FR2, and FR3 were mapped, respectively, to the historian-persistence analysis, the controlled functional scenarios, and the bidirectional-configuration tests. Operational-recovery and edge-resource tests provided complementary evidence regarding the behavior of the implemented architecture.

### 3.3. Supervisory Architecture and Implementation

The implemented architecture was organized into four layers: field, communication, edge server, and visualization, as shown in Figure 3. These layers delimited the allocation of computational responsibilities and the interfaces between the physical process, embedded acquisition, supervisory processing, and human–machine interaction. Process data followed the forward path from the field layer to the human–machine interface (HMI), whereas configuration commands and their acknowledgments followed the reverse path. This organization provided the implementation mechanisms for the separation between acquisition and supervision (AR1) and for the four-layer modular structure (AR7).

The field layer included the eleven measurement channels, their signal-conditioning circuits, and an ESP32 microcontroller. Pressure, pH, and electrical-conductivity signals were acquired through ADC1 analog inputs, while flow-meter pulses were processed asynchronously through digital interrupts. The temperature sensors used independent 1-Wire buses. The firmware converted the acquired signals into engineering units using the experimentally determined calibration coefficients, applied the required filtering and temperature compensation, and produced auxiliary flow-meter information, including accumulated volume and signal-failure indicators. The process variables and auxiliary states were delivered to the communication layer at a nominal publication rate of 1 Hz. Acquisition and primary signal processing remained operationally detached from the supervisory server, as specified by AR1.

The communication layer used a local Wi-Fi network and MQTT over TCP/IP. The ESP32 and the supervisory backend connected to a Mosquitto broker through the PubSubClient and Paho MQTT libraries, respectively. Each process variable was published in an individual topic under the common hierarchy planta/agua/esp32_01/sensores/. Periodic measurements were transmitted as scalar payloads using QoS 0 without broker retention, and their timestamps were assigned by the backend upon receipt. Configuration messages used JSON payloads, QoS 1, and broker retention, and were exchanged through separate set and status topics. This publish–subscribe arrangement implemented communication decoupling (AR3), while the topic hierarchy and standardized MQTT, TCP/IP, and JSON interfaces provided an architectural basis for extensibility (AR4) and supported interoperability (AR5).

The edge-server layer was deployed on a Raspberry Pi 3B+ and comprised the Mosquitto broker, a Python 3.11.2 backend implemented with Flask, and a local SQLite database. The backend was executed continuously as a systemd service and maintained a central configuration structure for the eleven tags. For each one, this structure associated the instrument description, engineering unit, MQTT topic, physical-validity criteria, operating limits, alarm criticality, functional dependencies, and participation in the determination of the global process state. This configuration served as the common source for state processing, alarm evaluation, historical persistence, diagnosis, and the REST resources consumed by the HMI.

After receiving an MQTT message, the backend identified the tag from its topic, converted the payload to the expected numerical type, assigned the reception timestamp, and updated the current measurement state. Each channel was evaluated for communication timeout, physical validity, operating range, local failure, and, where applicable, temperature dependency. The resulting supervisory states comprised NORMAL, OFFLINE, FAILURE, INVALID, HELD, HIGH, and LOW. The global process state provided additional context for this classification: range-based high and low conditions were suppressed while the RO system was stopped, whereas communication, validity, local-failure, and temperature-dependency evaluations remained active. Thus, the backend processed measurements together with their operational context rather than treating them as isolated numerical values.

Alarm management was based on transitions between consecutive supervisory states. When a new abnormal condition was detected, the previous occurrence, if present, was closed and a new alarm instance was created with its own priority, start time, and acknowledgment (ACK) state. Operator acknowledgment was received through an HTTP POST request and persisted without clearing the underlying condition. Only after a subsequent state transition was an alarm closed. SQLite operated in write-ahead logging mode (WAL) and stored sensor history, alarm occurrences, internal system states, sensor coefficients, and operational events. Measurements were persisted when a configured maximum interval elapsed, when their variation exceeded the corresponding threshold, or when their supervisory state changed. Sensor records were subject to a 90-day retention period, whereas alarm, coefficient, and system-event records were retained until operator intervention. These mechanisms implemented historical storage (FR1).

The visualization layer was implemented as a browser-based multipage HMI using HTML, CSS, and JavaScript. Overview, instrumentation, alarm, trend, diagnostic, and configuration functions were provided by the pages and their corresponding tabs. The browser accessed current values, historical data, alarms, diagnostics, and configuration resources exclusively through the Flask REST API. At no time did it connect directly to either the MQTT broker or SQLite. HTTP responses used JSON, whereas selected historical and alarm records could be exported as CSV files. Interface polling was separated from field acquisition and backend processing: current process information was updated every 2 s, alarm and trend information every 5 s, and infrastructure and configuration information every 10 s. This interface separation supported low coupling (AR2), while the aggregation of supervisory services in a single HMI implemented integrated supervision (FR2).

Remote configuration followed a complete bidirectional sequence. A parameter submitted through the HMI was first validated by the backend against its permitted domain, versioned, marked as pending, and published to the ESP32 as part of a complete set of 21 calibration coefficients. The firmware verified the presence and type of all required parameters and applied the package only when the complete validation succeeded. It then published a status message indicating success or failure, and the backend updated the persistent application state accordingly. Consequently, publication of a configuration message was not interpreted as evidence that the parameters had been applied at the field device. This confirmation mechanism implemented remote configuration (FR3). The use of Mosquitto, Python 3.11.2/Flask, SQLite, and standard web technologies provided the central supervisory functions without a proprietary SCADA runtime, supporting AR6.

### 3.4. Functional Validation Procedure

The validation addressed the supervisory architecture rather than the metrological performance of the individual COTS sensors. Three complementary procedures were used: architectural inspection, retrospective analysis of operational records, and controlled functional testing. This combination allowed structural properties and observable supervisory functions to be evaluated against the system-level requirements defined in Table 2; the correspondence between validation procedures and requirements is summarized in Table 3.

Architectural inspection examined the ESP32 firmware, MQTT clients and topics, Mosquitto configuration, supervisory backend, systemd service, SQLite schema, REST API, and HMI. The allocation of responsibilities and the interfaces between components were compared with AR1–AR7. Extensibility was assessed qualitatively by examining whether additional tags, field devices, and supervisory functions could be incorporated through the existing interfaces and configuration structures. Because no new tag or device was added specifically for this assessment, AR4 was supported by architectural inspection but was not experimentally validated.

Operational records from the eleven measurement channels, covering experimental sessions conducted from 28 May to 23 June 2026, were used to evaluate historical persistence. Because acquisition was discontinuous, a new session was defined whenever the interval between consecutive records exceeded 10 min. The analysis verified tag coverage, the presence of values, states, units, and timestamps, as well as temporal ordering, missing and duplicate records, and variable–unit consistency. Within each session, the median, 95th percentile, and maximum inter-record intervals were determined and compared with the historian configuration.

Functional validation combined four evidence layers: scripted MQTT input, REST API state, SQLite persistence, and HMI screenshots. Controlled scenarios covered normal operation, invalid values, operating-limit violations, communication loss, simultaneous thermal-dependency loss, normalization, alarm ACK, stopped-state behavior, and valid and invalid configuration changes. For each test, the initial condition, applied input, expected response, observed response, and timestamps were recorded. The six HMI pages were also examined for data updating and consistency among values, units, states, and timestamps. Alarm acknowledgment, historical retrieval and export, trends, diagnostics, and controlled parameter modification were also inspected.

The abnormal scenarios were generated as deterministic software inputs at the MQTT communication boundary. They evaluated the response of the supervisory software to defined values and loss of publications; they did not reproduce physical sensor disconnection, sensor drift, hardware degradation, hydraulic disturbances, or automatic closed-loop process control.

Operational recovery was evaluated by terminating the backend service 10 times, interrupting the Mosquitto broker 10 times, blocking ESP32 communication for 60 s in 10 repetitions, and completely restarting the Raspberry Pi three times. A recovery repetition was considered successful when the affected service or data path was restored and the applicable HTTP, MQTT, historian-progression, service-state, and SQLite integrity checks were satisfied.

Edge-resource utilization was measured at 1 s intervals during a controlled 600 s workload comprising 11 MQTT publications per second, requests to /api/dados every 2 s, requests to /api/alarmes every 5 s, and requests to /overview every 10 s. The recorded metrics included recovery time, CPU utilization, RAM utilization, backend resident set size, storage variation, HTTP and MQTT error counts, historian growth, and SQLite integrity. CPU and memory measurements were treated descriptively because no a priori capacity thresholds were defined. These complementary tests characterized implementation recovery and resource use but did not constitute additional architectural requirements.

Each requirement was classified as met when all predefined criteria and supporting evidence were obtained, partially met when only part of the criterion was demonstrated, or not demonstrated when the available evidence was insufficient. Cybersecurity assessment, formal usability testing, ergonomic evaluation, and scalability benchmarking were not considered in the validation scope.

Generative AI tools were used during manuscript preparation to assist with language revision, structural organization, drafting refinement, and consistency checks. These tools were not used to generate experimental data, execute validation tests, or calculate the reported results. All AI-assisted content was critically reviewed, technically verified, and edited by the authors.

## 4. Results

### 4.1. Integrated Operation and Historical Persistence

The historian contained 190,840 measurement records acquired from the eleven configured tags between 28 May and 23 June 2026. The interval between the first and last records was 616.6 h. However, this period included interruptions and was not interpreted as continuous RO-system operation. Using a gap greater than 10 min to delimit sessions, 60 aggregated acquisition sessions were identified. The longest session lasted 9 h 8 min 20 s and contained 25,973 records.

All configured measurement channels were represented in the retrieved data. The observed persistence intervals remained close to the historian configuration, as summarized in Table 4. Pressure records presented a median interval of 6 s, whereas the analytical variables presented medians between 15 and 16 s. Flow-rate and temperature records presented medians between 30 and 31 s. In all groups, the 95th percentile exceeded the corresponding median by no more than 2 s. These intervals characterize the persistence behavior of the historian rather than the ESP32 publication period because storage could also be triggered by value variation or measurement-state changes.

Of the 190,840 measurement records, 119,075 (62.40%) were stored with a normal condition, 41,265 (21.62%) with an operational-limit violation, 27,910 (14.62%) as invalid, and 2590 (1.36%) as offline. Twelve records contained no numerical value, all of which were associated with the offline condition. No invalid timestamps or variable–unit inconsistencies were identified.

A total of 982 same-tag records occurred within the same one-second timestamp resolution. Among them, 941 differed in value or state and were consistent with independent persistence triggers, whereas 41 were exact duplicates, corresponding to 0.02% of the measurement dataset. The alarm history contained 11,142 records covering all eleven tags, including 90 records marked as acknowledged. No required alarm fields were missing.

### 4.2. Controlled Functional Validation

Normal-operation input produced 11/11 online and normal tags, classified the RO system as OPERATING, and produced no publication errors over 3036 MQTT messages. The controlled tests subsequently exercised invalid-value, operating-limit, communication-loss, thermal-dependency, normalization, alarm-acknowledgment, and stopped-state conditions. The verified responses comprised instrument-state assignment, alarm generation and persistence, dependency handling, diagnostic reporting, and system-state classification.

Figure 4 presents the normal-operation baseline and representative HMI responses for five of the controlled scenarios. The operating-limit and normalization tests, which are not included as figure panels, are reported in Table 5 together with the remaining validation results.

The thermal-dependency and alarm-acknowledgment batch completed 1406 publication cycles and 14,686 MQTT messages without a publication error. During the stopped-state test, the accumulated-volume fields were neither published nor modified.

### 4.3. Bidirectional Configuration Validation

Bidirectional configuration was evaluated using the pulses-per-liter (PPL) coefficient of FT-401. The active value was changed from 409.0 to 409.1 pulses L^−1^ through the HMI. After the request was validated by the backend, the updated coefficient was stored with an incremented version and the complete coefficient package was transmitted to the ESP32 via MQTT. The update remained pending until the ESP32 returned a status message confirming its application. Afterwards, the coefficient was marked as APPLIED, as shown in Figure 5a,b.

After the valid-update test, the FT-401 coefficient was restored to its initial value of 409.0 pulses L^−1^, and the restoration was also confirmed by the ESP32. An out-of-range value of 2001.0 pulses L^−1^ was then entered through the HMI. Because it exceeded the configured maximum of 2000.0 pulses L^−1^, the update was rejected during validation and the active coefficient remained the same, as documented in Figure 5c,d.

These results verified both the bidirectional application of valid parameters and the preservation of the active configuration when an invalid update was requested.

### 4.4. Operational Recovery and Edge-Server Resource Utilization

Controlled interruption tests were used to characterize the recovery of the backend service, MQTT broker, ESP32 communication path, and complete edge server. All repetitions satisfied their applicable recovery criteria. Table 6 summarizes the observed times. Broker-service recovery and restoration of the complete data path to the REST API are reported separately because they represent different stages of the supervisory communication chain. Similarly, communication-loss detection and data-flow recovery after removal of the ESP32 traffic block are presented as distinct measurements.

During the ten controlled interruptions of the ESP32–broker communication path, all eleven measurement tags were classified as OFFLINE, while the REST API and HMI remained available. The median detection time of 9.863 s was consistent with the configured 10 s communication timeout. After the traffic block was removed, measurement updates and historical persistence resumed in all repetitions, with a median recovery time of 1.517 s. Backend and broker interruptions also resulted in automatic service recovery in all ten repetitions. Following the three complete Raspberry Pi restarts, the backend and Mosquitto services were active, MQTT communication was restored, the API and HMI returned HTTP status 200, and the SQLite integrity check returned ok. The comparatively wide 40.035–111.404 s restart range is reported descriptively because only three complete restarts were performed.

During the 600 s controlled resource-utilization test, 600 system samples were collected while 6600 MQTT messages and 480 HTTP requests were processed. No MQTT or HTTP errors were recorded. The historian increased by 471 records, and the SQLite integrity check returned ok. Mean, 95th-percentile, and maximum system CPU utilization were 8.425%, 10.445%, and 27.525%, respectively. For the backend process, the corresponding CPU values were 7.055%, 10.000%, and 21.963%. Mean RAM utilization was 51.925%, with a maximum of 52.783%, whereas the backend resident set size remained between a mean of 39,867.6 KiB and a maximum of 39,976 KiB. Disk occupancy increased from 65.0085% to 65.0143%, and the total SQLite footprint increased by 12,392 bytes, attributable to the write-ahead log.

The workload was generated locally at the MQTT broker and therefore characterizes the computational behavior of the edge server under controlled supervisory load rather than the performance of the physical sensors, Wi-Fi link, or complete acquisition chain. As only one workload level was examined and no previous capacity thresholds were defined, these measurements constitute a descriptive resource assessment rather than a scalability benchmark or experimental validation of AR4.

Taken together, the historian analysis, controlled functional scenarios, and bidirectional-configuration tests provided experimental evidence for the three functional requirements defined in the methodology: historical storage (FR1), integrated supervision (FR2), and remote configuration (FR3). The recovery and resource-utilization tests supplied complementary evidence about the implemented architecture but were not treated as additional system-level requirements. No inconsistencies were observed across the REST API, SQLite, alarm, diagnostic, and HMI evidence in the controlled scenarios in which these layers were jointly evaluated.

## 5. Discussion

The results demonstrate that an open edge-based supervisory architecture can integrate a set of functions commonly associated with SCADA platforms. The evidence obtained supports the three functional requirements: historical storage (FR1), integrated supervision (FR2), and remote configuration (FR3). AR1–AR3 and AR5–AR7 were supported primarily by inspection of the implemented allocation of responsibilities, interfaces, communication mechanisms, and layer organization. AR4 was supported only as an intended architectural property and was not experimentally validated through the addition of new tags, field devices, or supervisory modules.

The historian results indicate that the implemented persistence mechanism preserved the information required to reconstruct the monitored conditions. All eleven tags were represented among the 190,840 retrieved records, with their values, states, units, and timestamps available for analysis. The observed inter-record intervals remained close to the configured time-trigger thresholds, although these intervals do not represent the ESP32 acquisition or MQTT publication rate because persistence could also be initiated by value variation or supervisory-state transitions. The 41 exact duplicates represented only 0.02% of the dataset and did not compromise tag coverage or temporal analysis. Nevertheless, their presence shows that simultaneous persistence triggers can generate redundant records. These duplicates could be addressed by transaction-level deduplication if stricter storage efficiency were required.

The distribution of historical states should not be interpreted as an estimate of the normal availability or performance of the RO system. The dataset included records from interrupted experimental sessions and deliberately imposed abnormal conditions. In particular, the proportions of invalid, offline, and operating-limit records partly reflect the validation procedures themselves. The relevant result is therefore the preservation of the state associated with each measurement rather than the relative frequency of each one. This treatment is consistent with the view that industrial data quality depends on contextual information, traceability, and lifecycle management and cannot be inferred from numerical availability alone [22,31].

The controlled scenarios demonstrated that the architecture preserved the distinction between communication availability, physical validity, operating range, thermal dependency, and global system state. These distinctions prevented unavailable or physically invalid measurements from being represented as valid process values. They also allowed range-based alarms to be suppressed when the RO system was stopped while communication, validity, and dependency diagnoses remained active. This behavior is consistent with lifecycle-based alarm management, in which the originating condition, operator acknowledgment, and return to normal are treated as distinct elements [21,32]. Alarm acknowledgment was persisted without removing the underlying cause, and the alarm occurrence was closed only after its normalization. This separation between condition, acknowledgment, and closure is operationally relevant because an operator action does not imply that the originating condition has ceased. This behavior provided information that may support operator situational awareness in a dynamic process [33].

Bidirectional configuration provided an additional form of cross-layer traceability. A coefficient was classified as APPLIED only after the ESP32 confirmed application of the transmitted package. Therefore, successful validation and MQTT publication by the backend were not treated as sufficient evidence of execution at the field device. Conversely, the out-of-range coefficient was rejected before transmission, and the active value remained unchanged. This sequence distinguishes a requested configuration, a transmitted configuration, and a physically confirmed configuration, reducing the risk of inconsistencies between the supervisory database and the embedded device.

The present results complement previous open supervisory implementations by extending the validation scope beyond technological feasibility. Uddin et al. demonstrated accessible local supervision of a community RO system [5]. Kafrashi et al. emphasized local availability and bidirectional control under intermittent Internet connectivity [10], while Poyatos-Bakker et al. addressed modularity and interoperability in a demonstrative-scale desalination system [11]. The additional evidence provided here concerns cross-layer coherence: measurement states, alarm occurrences, diagnostics, historical records, HMI information, and configuration acknowledgments were verified across the field, communication, edge-server, and visualization layers. This difference concerns the scope of integration and validation reported in each study and should not be interpreted as evidence of technological superiority.

The recovery tests indicate that the main services resumed operation automatically after controlled interruptions. Median recovery times were 6.384 s for the backend API, 1.312 s for restoration of the MQTT data path to the REST API, and 1.517 s for measurement updates after removal of the ESP32–broker communication block. Recovery after a complete Raspberry Pi restart exhibited greater variation, with a median of 90.604 s and an observed range of 40.035–111.404 s. All tested repetitions satisfied the applicable service, communication, persistence, and database-integrity checks. However, these results characterize recovery under controlled local conditions and should not be converted into availability or reliability estimates.

The edge-server resource measurements show that the imposed workload was processed without MQTT or HTTP errors and without database-integrity failure. System CPU utilization remained below 27.525%, backend CPU utilization remained below 21.963%, and RAM utilization had a mean of 51.925% and a maximum of 52.783%. These results indicate that the tested Raspberry Pi configuration accommodated the specific workload of 11 MQTT publications per second and 480 HTTP requests over 600 s. They do not demonstrate capacity under larger numbers of devices, higher publication rates, concurrent users, or prolonged operation. Because the workload was generated locally at the broker, it also excluded the physical sensors and Wi-Fi acquisition path. The resource test must be interpreted solely as a bounded implementation check rather than a scalability benchmark.

The principal limitations arise from the experimental scope. The architecture was evaluated on a single RO system, one edge-server configuration, and one local network. The COTS sensors were used as process-data sources and were not evaluated as reference-grade instruments; consequently, the study does not establish metrological performance or improvements in RO separation efficiency. Cybersecurity, formal operator usability, alarm rationalization with operating personnel, and ergonomic conformity were also outside the validation scope. Future work should therefore evaluate sustained operation, authenticated and encrypted communication, multiple concurrent field devices, increased tag and user loads, and the addition of new modules to provide direct experimental evidence for AR4. Formal usability studies and comparison with reference instrumentation would address limitations that cannot be resolved through software testing alone.

## 6. Conclusions

This study developed and functionally validated a four-layer open supervisory architecture for an experimental RO system, integrating field acquisition, MQTT-based communication, supervisory processing, historical persistence, and a browser-based HMI. Its main contribution lies in the integrated implementation and cross-layer validation of these services rather than in the isolated use of its hardware, communication protocols, or software components.

The historian contained 190,840 records covering all eleven configured tags and preserved the values, states, units, and timestamps required for historical analysis. Controlled functional scenarios (including normal operation, invalid values, operating-limit violations, communication loss, and alarm acknowledgment) demonstrated the system’s coherent responses across layers. Bidirectional configuration distinguished parameter validation, transmission, embedded application, and ESP32 confirmation.

The validation provided evidence that the functional requirements for historical storage (FR1), integrated supervision (FR2), and remote configuration (FR3) were met under the examined conditions. AR1–AR3 and AR5–AR7 were supported by inspection of the implemented architecture, including role allocation, interfaces, communication mechanisms, and the underlying technology stack. AR4, interpreted as extensibility, was supported only as an intended architectural property and was not experimentally validated through the addition of new tags, devices, or supervisory modules. Although the recovery and resource-utilization tests supplied complementary implementation evidence, they did not constitute a scalability benchmark.

The conclusions are limited to the experimental system, hardware configuration, network, controlled scenarios, and workload examined. The COTS sensors were used as process-data sources, and their metrological performance was not evaluated.

Collectively, these results support the feasibility of the proposed architecture for integrated supervision of small-scale experimental RO systems under the tested conditions. This evaluation, however, did not address cybersecurity, ergonomic conformity, or performance under increasing numbers of devices. Future work should therefore focus on these aspects to establish the architecture’s readiness and robustness for broader deployment.

## Figures and Tables

**Figure 1:**
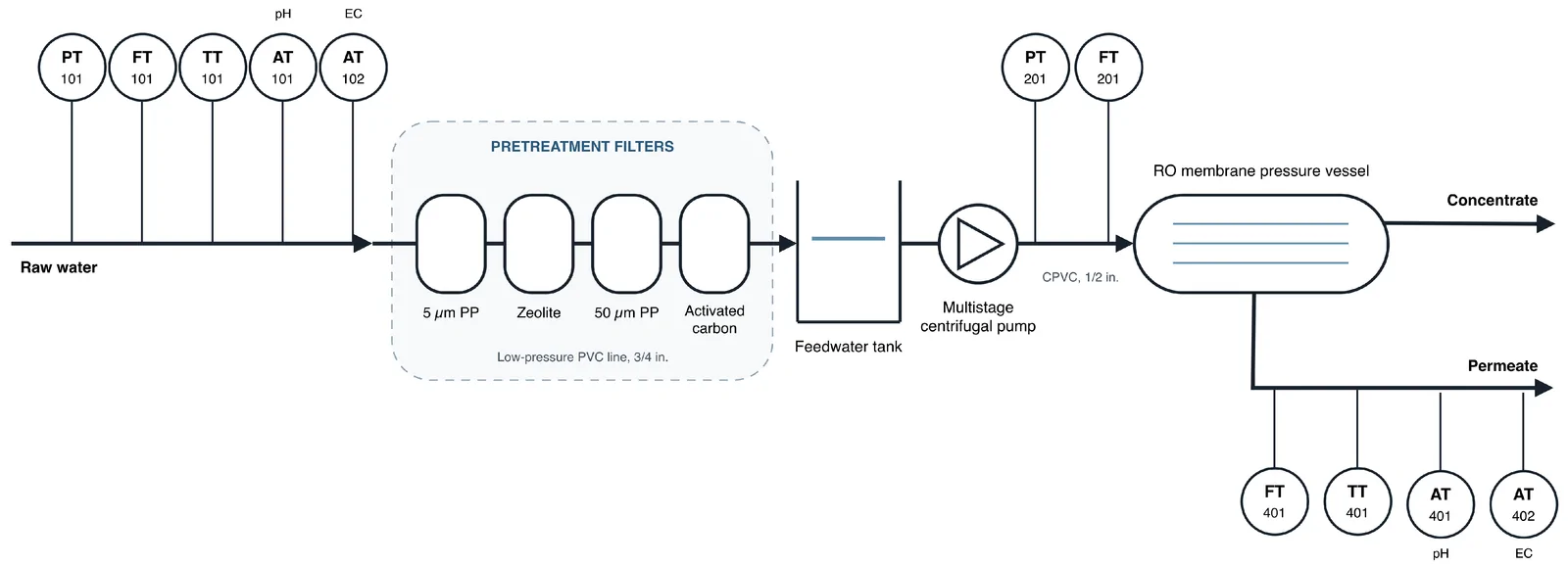
Hydraulic arrangement of the experimental RO system and locations of the measurement channels.

**Figure 2:**
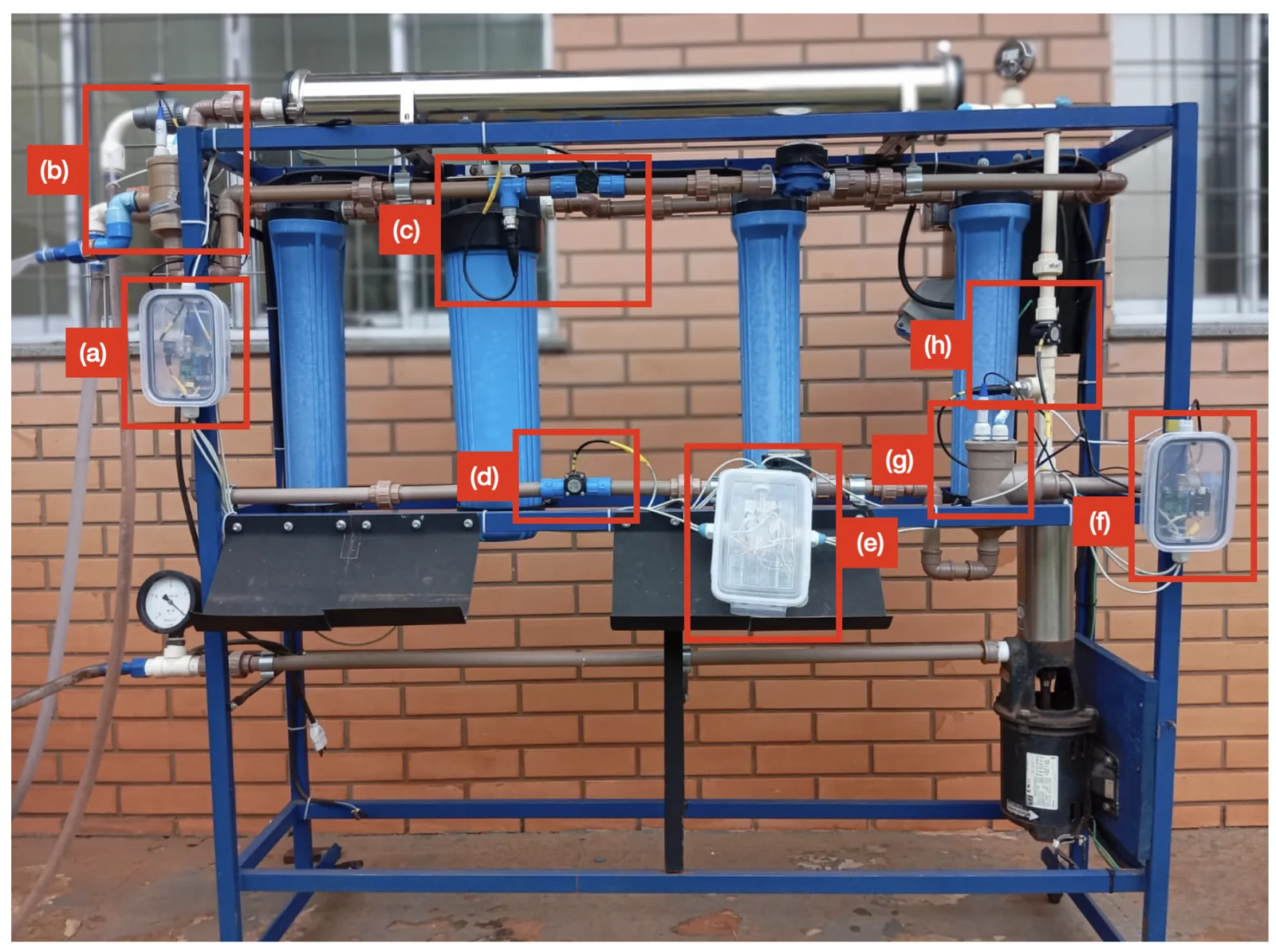
Instrumentation installed in the experimental reverse-osmosis system: (**a**) raw-water sensor interface and signal-conditioning enclosure; (**b**) TT-101, AT-101, and AT-102; (**c**) PT-101 and FT-101; (**d**) FT-401; (**e**) ESP32-based acquisition node; (**f**) permeate sensor interface and signal-conditioning enclosure; (**g**) TT-401, AT-401, and AT-402; and (**h**) PT-201 and FT-201.

**Figure 3:**
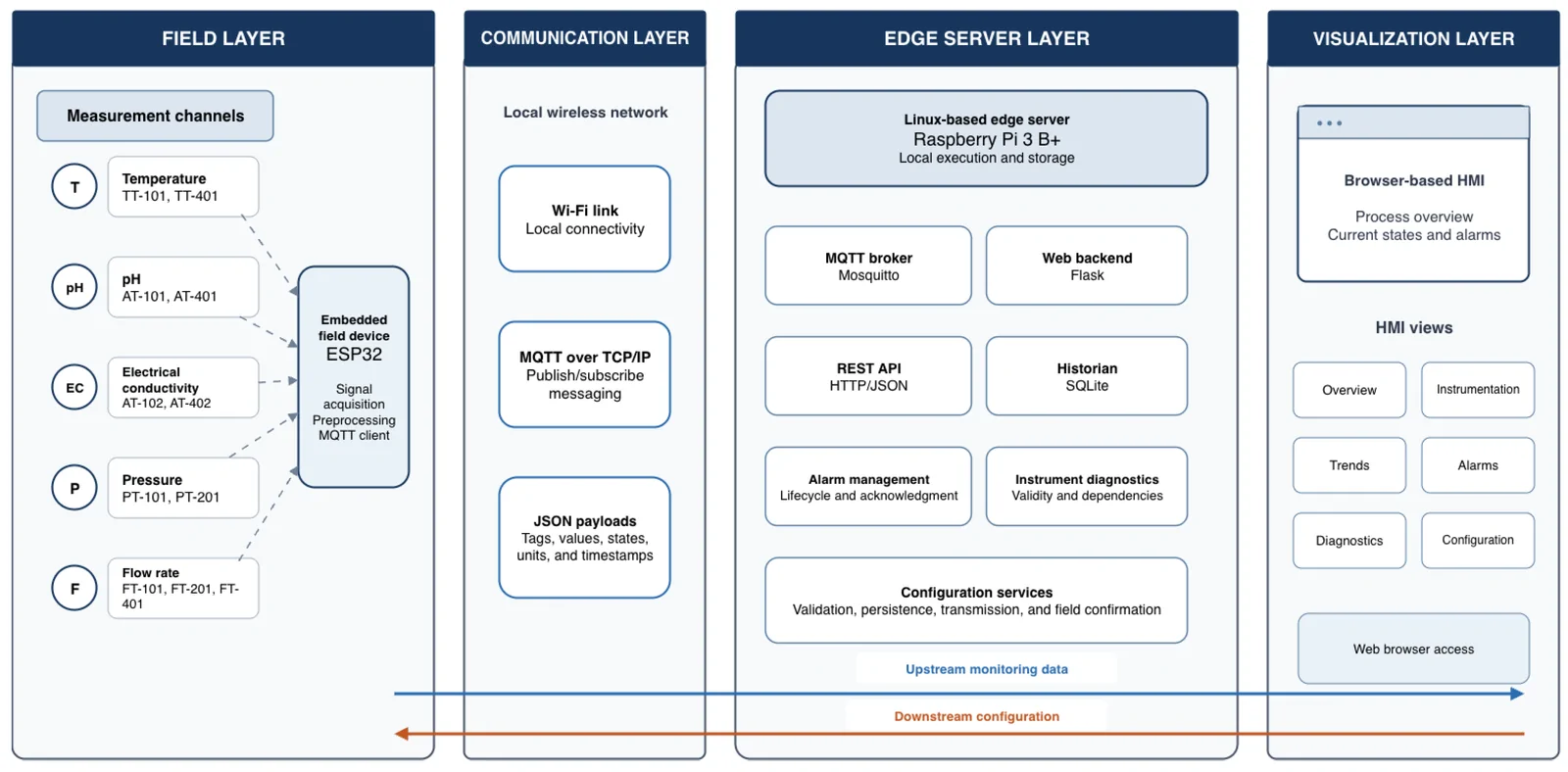
Implemented four-layer supervisory architecture and bidirectional information flows.

**Figure 4:**
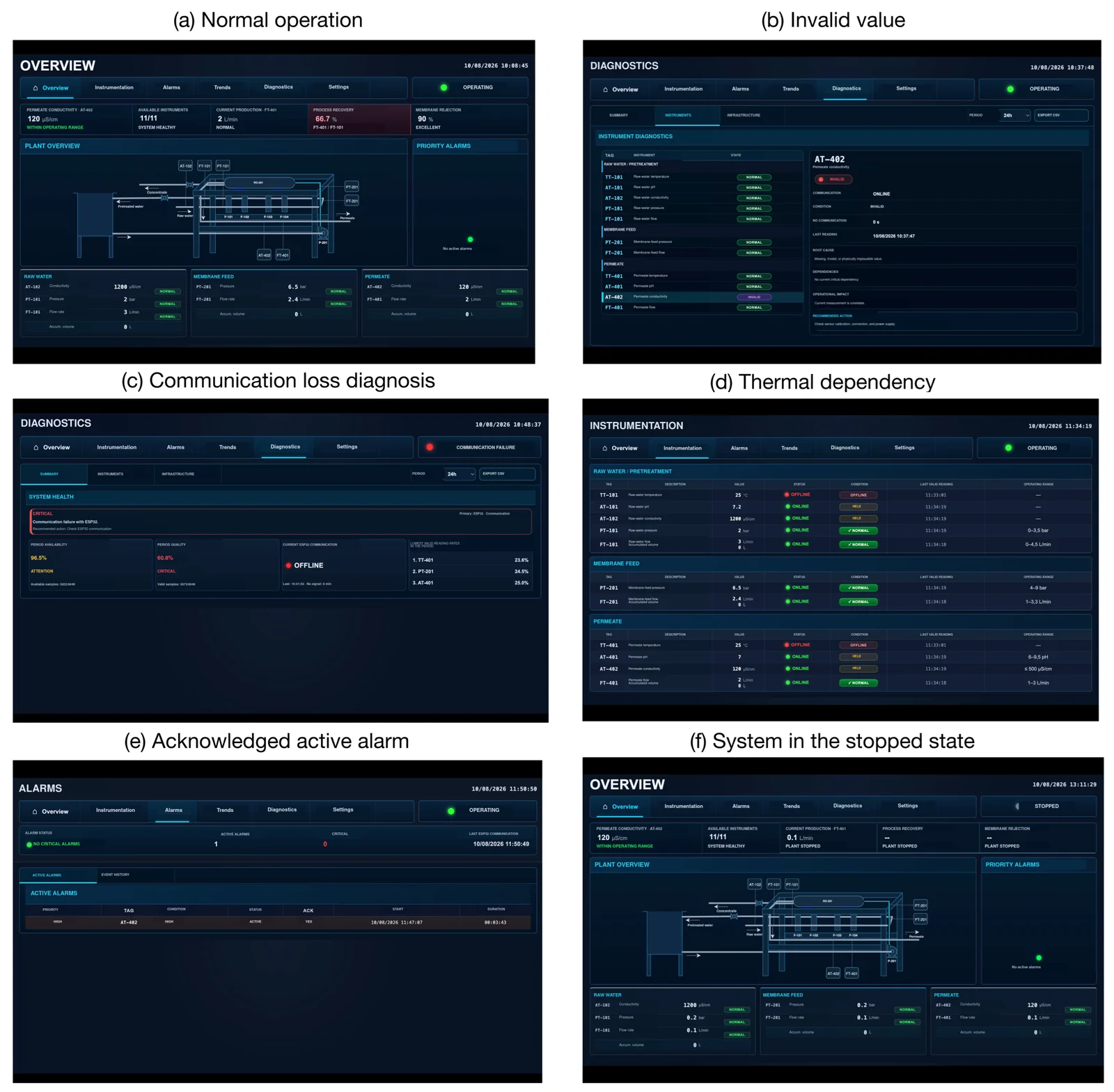
Representative HMI responses during controlled functional validation: (**a**) normal operation, showing *OPERATING*, *WITHIN OPERATING RANGE*, and *SYSTEM HEALTHY*; (**b**) invalid AT-402 input, marked *INVALID*, with communication maintained; (**c**) complete publication loss, reported as *COMMUNICATION FAILURE* and *CRITICAL*; (**d**) temperature-channel loss, with the dependent analytical channels marked as *HELD*; (**e**) acknowledged active high-priority alarm; and (**f**) stopped RO system, indicated by *STOPPED*, with all 11 instruments available and no priority alarms.

**Figure 5:**
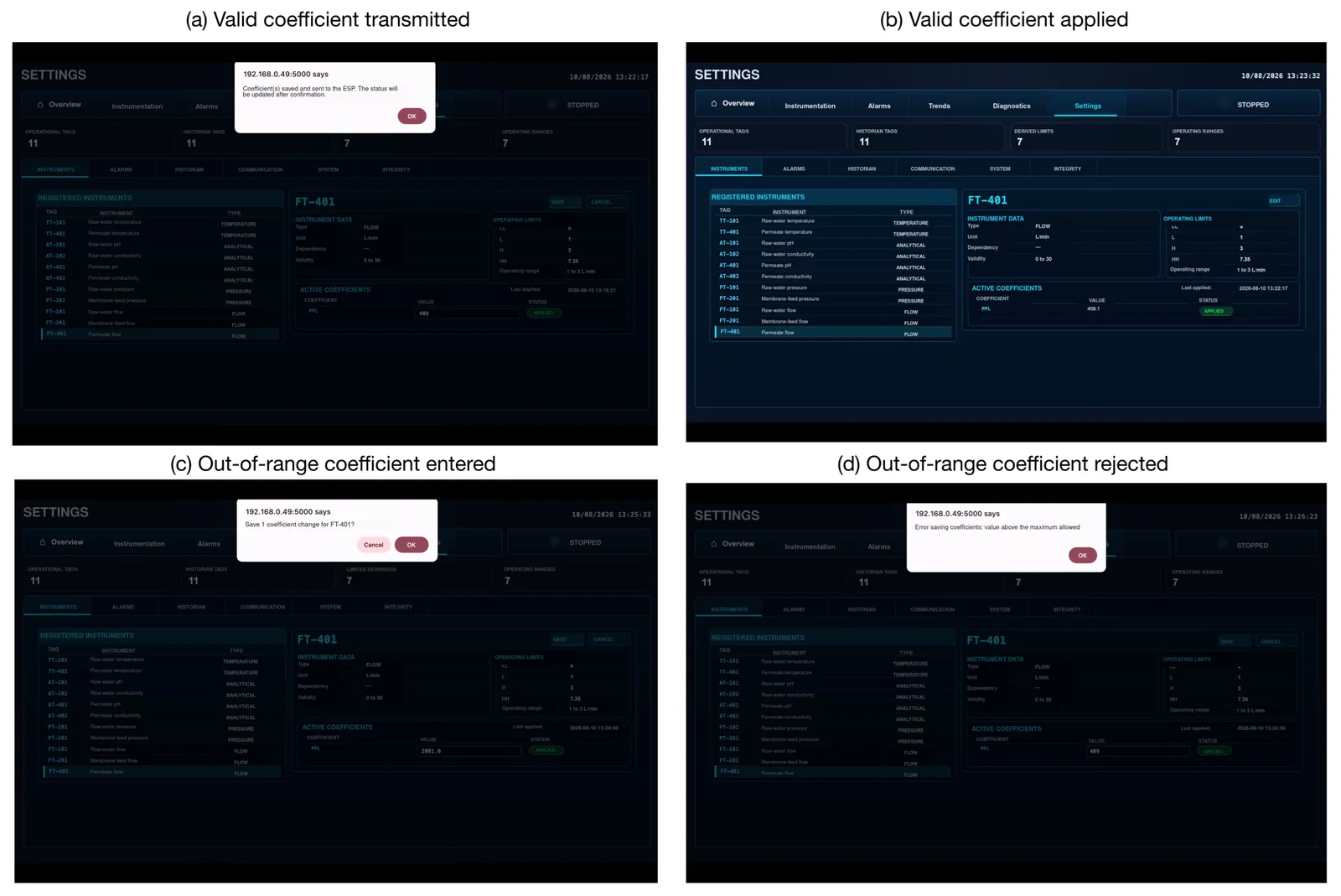
HMIevidence from the bidirectional configuration validation. The RO system remained in the *STOPPED* state throughout the tests: (**a**) valid FT-401 coefficient update saved and transmitted to the ESP32, with the notification indicating that the status would be updated after embedded confirmation; (**b**) updated value of 409.1 pulses L^−1^ identified as *APPLIED* after confirmation by the ESP32; (**c**) out-of-range value of 2001.0 pulses L^−1^ entered and submitted for confirmation after the active coefficient had been restored to 409.0 pulses L^−1^; and (**d**) update rejected with a notification that the value exceeded the maximum permitted limit, while the active coefficient remained at 409.0 pulses L^−1^.

**Table 1:** Measurement channels of the experimental reverse osmosis system.

Measured Quantity	Tags	No. of Channels	Unit
Pressure	PT-101, PT-201	2	bar
Flow rate	FT-101, FT-201, FT-401	3	L min^−1^
Temperature	TT-101, TT-401	2	°C
pH	AT-101, AT-401	2	–
Electrical conductivity (EC)	AT-102, AT-402	2	μS cm^−1^
**Total**	–	**11**	–

**Table 2:** Functional and architectural requirements of the supervisory platform.

ID	Requirement	Design Implication
AR1	Separation between acquisition and supervision	Field acquisition and edge supervision
AR2	Low coupling between modules	Explicit module interfaces
AR3	Communication decoupling	Broker-mediated data exchange
AR4	Extensibility	Addition of tags, devices, and functions via the existing interfaces
AR5	Interoperability	Standard protocols and data formats
AR6	Use of open technologies	No proprietary SCADA dependency
AR7	Modularity	Four-layer functional organization
FR1	Historical storage	Measurements, events, and alarms
FR2	Integrated supervision	Unified supervisory services
FR3	Remote configuration	Bidirectional parameter management

**Table 3:** Validation procedures and associated requirements.

Procedure	Principal Verification	Requirements
Architectural inspection	Role allocation, interfaces, modularity, interoperability, openness, and qualitative extensibility assessment	AR1–AR3, AR5–AR7; AR4 (qualitative)
Operational-record analysis	Persistence, retrieval, coverage, temporal organization, and structural integrity of historical records	FR1
Controlled functional testing	Measurement states, alarms, HMI functions, diagnosis, and integrated supervisory operation	FR2; AR2–AR3
Bidirectional configuration testing	Validation, transmission, application, acknowledgment, and persistence of configuration parameters	FR3

**Table 4:** Configured time-trigger thresholds and observed inter-record intervals within the experimental sessions.

Variable Group	Tags	Configured Time-Trigger Threshold (s)	Range of Tag-Specific Medians (s)	Tag-Specific 95th Percentile (s)
Pressure	PT-101, PT-201	5	6	7
pH and electrical conductivity	AT-101, AT-102, AT-401, AT-402	15	15–16	17
Flow rate and temperature	FT-101, FT-201, FT-401, TT-101, TT-401	30	30–31	32

**Table 5:** Results of the controlled functional validation.

Scenario	Imposed Condition	Verified Response
Invalid value	AT-402 = 2100 μS cm^−1^, beyond the physical domain	Communication remained online while the measurement was classified as INVALID; alarm generation and instrument diagnosis
Operating limit	AT-402 = 600 μS cm^−1^, above H = 500 μS cm^−1^	HIGH state and active high-priority alarm
Communication loss	Suspension of all 11 controlled publications	Eleven OFFLINE tags, critical alarms, and infrastructure diagnosis
Thermal dependency	Suspension of TT-101 and TT-401 publications	Two temperature tags OFFLINE; four analytical tags HELD; detection after 10.88 s for a configured timeout of 10 s
Normalization	Temperature publications resumed	Six affected alarm occurrences closed within 2.58 s, and the corresponding tags returned to normal states
Alarm acknowledgment	HMI acknowledgment of an active AT-402 high alarm	ACK persistence in the API and SQLite after the HMI was reloaded and after the occurrence was closed; alarm remained active until normalization
Stopped-state condition	Pressure and flow-rate values below the stopped-state thresholds	RO system classified as STOPPED; 11/11 tags online; no active range alarms

**Table 6:** Observed recovery and communication-loss detection times.

Intervention and Measured Response	Successful Repetitions	Minimum (s)	Median (s)	Maximum (s)
Backend service interruption: API recovery	10/10	6.359	6.384	6.482
Mosquitto interruption: broker-service recovery	10/10	0.217	0.225	0.237
Mosquitto interruption: restoration of data flow to the REST API	10/10	1.080	1.312	1.344
ESP32–broker interruption: OFFLINE detection	10/10	9.355	9.863	10.141
ESP32–broker interruption: data-flow recovery	10/10	1.166	1.517	2.207
Complete Raspberry Pi restart: supervisory-service recovery	3/3	40.035	90.604	111.404

## Data Availability

The data and validation materials supporting the findings of this study are openly available in Zenodo at https://doi.org/10.5281/zenodo.21906111. The complete supervisory-system source code is not included because it contains deployment-specific configuration and is outside the scope of the deposited dataset.

## Abbreviations

The following abbreviations are used in this manuscript:

ACK	Alarm acknowledgment
HMI	Human–machine interface
IIoT	Industrial Internet of Things
MQTT	Message Queuing Telemetry Transport
RO	Reverse osmosis
SCADA	Supervisory control and data acquisition

## References

[1] Mirani A.A., Velasco-Hernandez G., Awasthi A., Walsh J. (2022). Key Challenges and Emerging Technologies in Industrial IoT Architectures: A Review. Sensors.

[2] Babayigit B., Abubaker M. (2024). Industrial Internet of Things: A Review of Improvements over Traditional SCADA Systems for Industrial Automation. IEEE Syst. J..

[3] Brad S., Murar M., Vlad G., Brad E., Popanton M. (2021). Lifecycle Design of Disruptive SCADA Systems for Waste-Water Treatment Installations. Sustainability.

[4] Dwarakanath B., Kalpana Devi P., Ranjith Kumar A., Metwally A.S.M., Ashraf G.A., Thamineni B.L. (2023). Smart IoT-Based Water Treatment with a Supervisory Control and Data Acquisition (SCADA) System Process. J. Water Reuse Desalin..

[5] Uddin S.U., Baig M.J.A., Iqbal M.T. (2022). Design and Implementation of an Open-Source SCADA System for a Community Solar-Powered Reverse Osmosis System. Sensors.

[6] Martínez R., Vela N., El Aatik A., Murray E., Roche P., Navarro J.M. (2020). On the use of an IoT integrated system for water quality monitoring and management in wastewater treatment plants. Water.

[7] Nuñez J., Benítez I., Rodríguez A., Díaz S., De Oliveira D. (2019). Tools for the Implementation of a SCADA System in a Desalination Process. IEEE Lat. Am. Trans..

[8] Alshehri M., Bhardwaj A., Kumar M., Mishra S., Gyani J. (2021). Cloud and IoT Based Smart Architecture for Desalination Water Treatment. Environ. Res..

[9] Dimitriou E., Loukatos D., Tampakakis E., Arvanitis K.G., Papadakis G. (2024). An Experimental Investigation of an Open-Source and Low-Cost Control System for Renewable-Energy-Powered Reverse Osmosis Desalination. Electronics.

[10] Kafrashi F., Golchin H., Iqbal M.T. (2025). Monitoring and Control of a Remote Hybrid Powered Reverse Osmosis Unit for McCallum, NL. J. Electron. Electr. Eng..

[11] Poyatos-Bakker A.R., Martínez-Roa A., Roca L., Palenzuela P., Gil J.D. (2025). Open-Source Web-Based SCADA System for a Demonstrative Desalination Plant. Jorn. Autom..

[12] Zaidi S.J., Saleem H. (2022). Reverse Osmosis Principles and System Components. Reverse Osmosis Systems: Design, Optimization and Troubleshooting Guide.

[13] Najdawi F.Z., Neptune K.T. (2022). Optimizing Reverse Osmosis Membrane Parameters through the Use of the Solution-Diffusion Model: A Review. Engineering.

[14] Tayeh Y.A. (2024). A Comprehensive Review of Reverse Osmosis Desalination: Technology, Water Sources, Membrane Processes, Fouling, and Cleaning. Desalin. Water Treat..

[15] Arora M., Maheshwari R.C., Jain S.K., Gupta A. (2004). Use of Membrane Technology for Potable Water Production. Desalination.

[16] Kucera J. (2023). Reverse Osmosis.

[17] Elyaakouby Y., El-Kordy A., Tilioua A. (2026). Analysis and Diagnostic of Reverse Osmosis Membrane Fouling Using Industrial Plant Data. Clean. Water.

[18] Omidi S.A., Baig M.J.A., Iqbal M.T. (2023). Design and Implementation of Node-RED-Based Open-Source SCADA Architecture for a Hybrid Power System. Energies.

[19] He W., Baig M.J.A., Iqbal M.T. (2024). An Open-Source Supervisory Control and Data Acquisition Architecture for Photovoltaic System Monitoring Using ESP32, Banana Pi M4, and Node-RED. Energies.

[20] (2015). Human Machine Interfaces for Process Automation Systems.

[21] (2022). Management of Alarm Systems for the Process Industries.

[22] Fu Q., Nicholson G.L., Easton J.M. (2024). Understanding Data Quality in a Data-Driven Industry Context: Insights from the Fundamentals. J. Ind. Inf. Integr..

[23] Wang C., Qiao J., Huang X., Song S., Hou H., Jiang T., Rui L., Wang J., Sun J. (2023). Apache IoTDB: A Time Series Database for IoT Applications. Proc. Acm Manag. Data.

[24] Faraj O., Megias D., Garcia-Alfaro J. (2025). Security Approaches for Data Provenance in the Internet of Things: A Systematic Literature Review. ACM Comput. Surv..

[25] Stouffer K., Pease M., Tang C., Zimmerman T., Pillitteri V.Y., Lightman S., Hahn A., Saravia S., Sherule A., Thompson M. (2023). Guide to Operational Technology (OT) Security.

[26] Seoudy H., Seoudy A., Fahmy A. (2024). Comparative Analysis of Centralized and Decentralized Control Systems for Nuwiebaa SWRO Desalination Plant. Results Eng..

[27] Laredj I.E., Abid M., Bessaoud K., Benhamida M. (2026). DigiRO: A Digital Model Framework for Reverse Osmosis Desalination Integrating Process Modeling and Industrial Control. Simul. Model. Pract. Theory.

[28] da Costa P.S. (2025). Sistema de Tratamento de Água Potável em Comunidades com Vulnerabilidade Hídrica Qualitativa. Master’s Thesis.

[29] (2024). Instrumentation and Control—Symbols and Identification.

[30] De Camargo E.T., Spanhol F.A., Slongo J.S., Da Silva M.V.R., Pazinato J., de Lima Lobo A.V., Coutinho F.R., Pfrimer F.W.D., Lindino C.A., Oyamada M.S. (2023). Low-cost water quality sensors for IoT: A systematic review. Sensors.

[31] Goknil A., Nguyen P., Sen S., Politaki D., Niavis H., Pedersen K.J., Suyuthi A., Anand A., Ziegenbein A. (2023). A Systematic Review of Data Quality in CPS and IoT for Industry 4.0. ACM Comput. Surv..

[32] (2016). Management of Alarm Systems for the Process Industries.

[33] Endsley M.R. (1995). Toward a Theory of Situation Awareness in Dynamic Systems. Hum. Factors.

